# Right Foot Trans Metatarsal Amputation Following COVID-19 Infection

**DOI:** 10.7759/cureus.27442

**Published:** 2022-07-29

**Authors:** Alvin D Sanhueza-Martinez, Sunjeet K Brar, Fabrice Yabit, Anika Risden, Minahal Asif, Frederick Tiesenga, Juaquito Jorge

**Affiliations:** 1 Medicine, Saint James School of Medicine, Arnos Vale, VCT; 2 Medicine, Aureus University School of Medicine, Oranjestad, ABW; 3 Surgery, West Suburban Medical Center, Chicago, USA

**Keywords:** chilblain's disease, diabetes, digital amputation, recurrent osteomyelitis, wound complication, covid-19 pneumonia, sars cov-2, corona, covid toes, covid-19

## Abstract

A 60-year-old male patient with a prior coronavirus disease 2019 (COVID-19) pneumonia diagnosis presented with a right foot ulcer. The ulcer progressed to osteomyelitis of his right fifth metatarsal with eventual amputation and resection of the affected digit. The infection recurred two months later and spread to the right fourth metatarsal and gangrene, leading to the amputation and partial metatarsal head resection of the fourth toe. A month later, the infection recurred for a second time and a decision to perform a right trans metatarsal amputation of the foot was evaluated to avoid further progression of the infection and the need for more invasive surgical intervention.

## Introduction

We present a case of a right trans metatarsal amputation due to gangrene in a patient with a history of coronavirus disease 2019 (COVID-19) pneumonia. It is essential to recognize that the COVID-19 virus does not only affect the lungs and the breathing capacity but there can also be many systemic complications. As a clinician, it is essential to rapidly detect these systemic symptoms to avoid any long-term complications. There has been an increase in cases linking COVID-19 to vascular and cutaneous manifestations such as in the case presented. This is a noteworthy case to present given a foot ulcer complication following a COVID-19 infection that led to a trans metatarsal amputation in a brief time. This case report aims to better understand the many and often rare COVID-19 virus complications and enhance patient care.

## Case presentation

**Figure 1:**
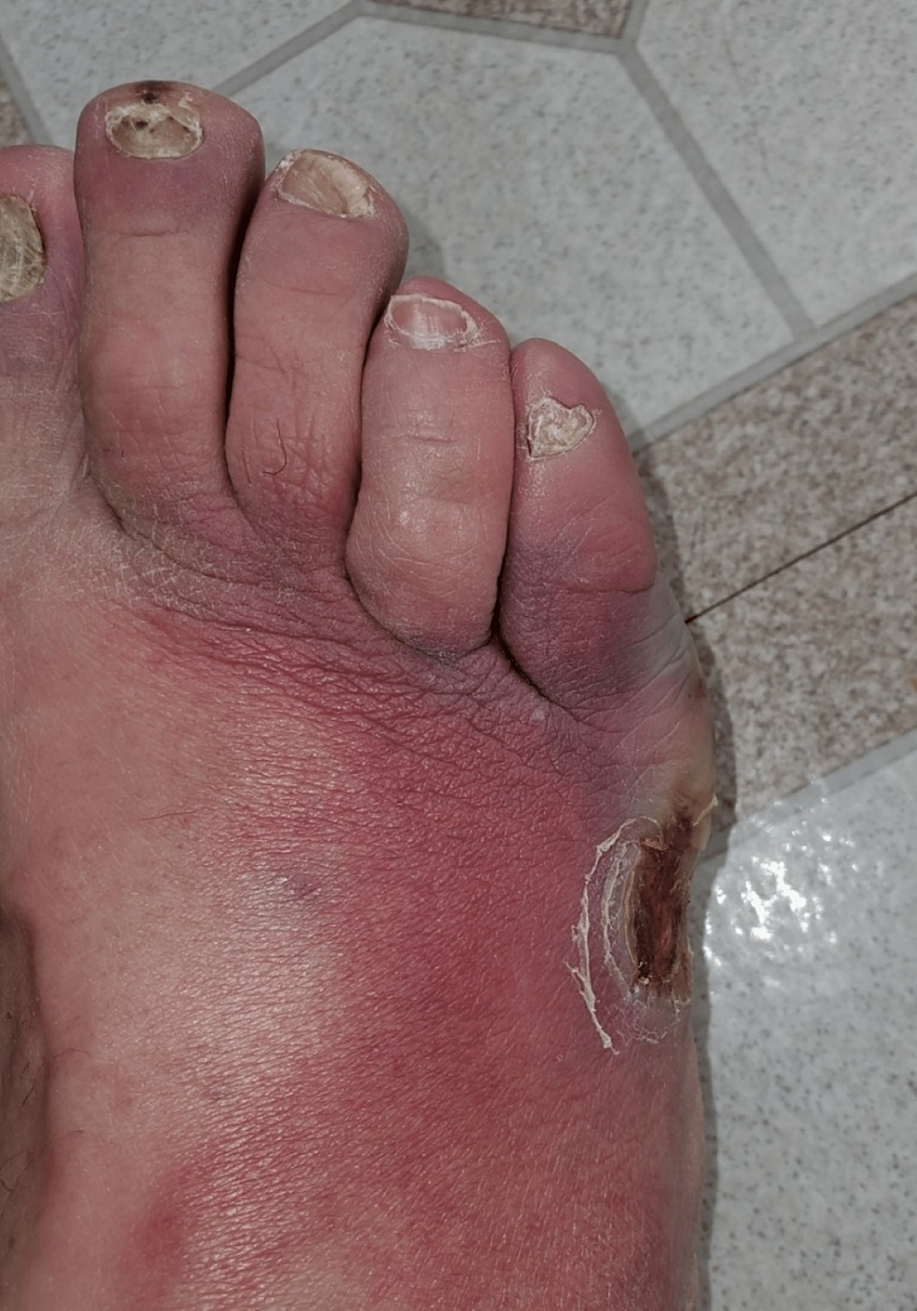
Superior view of the right foot prior to first surgery, erythematous ulcer.

**Figure 2:**
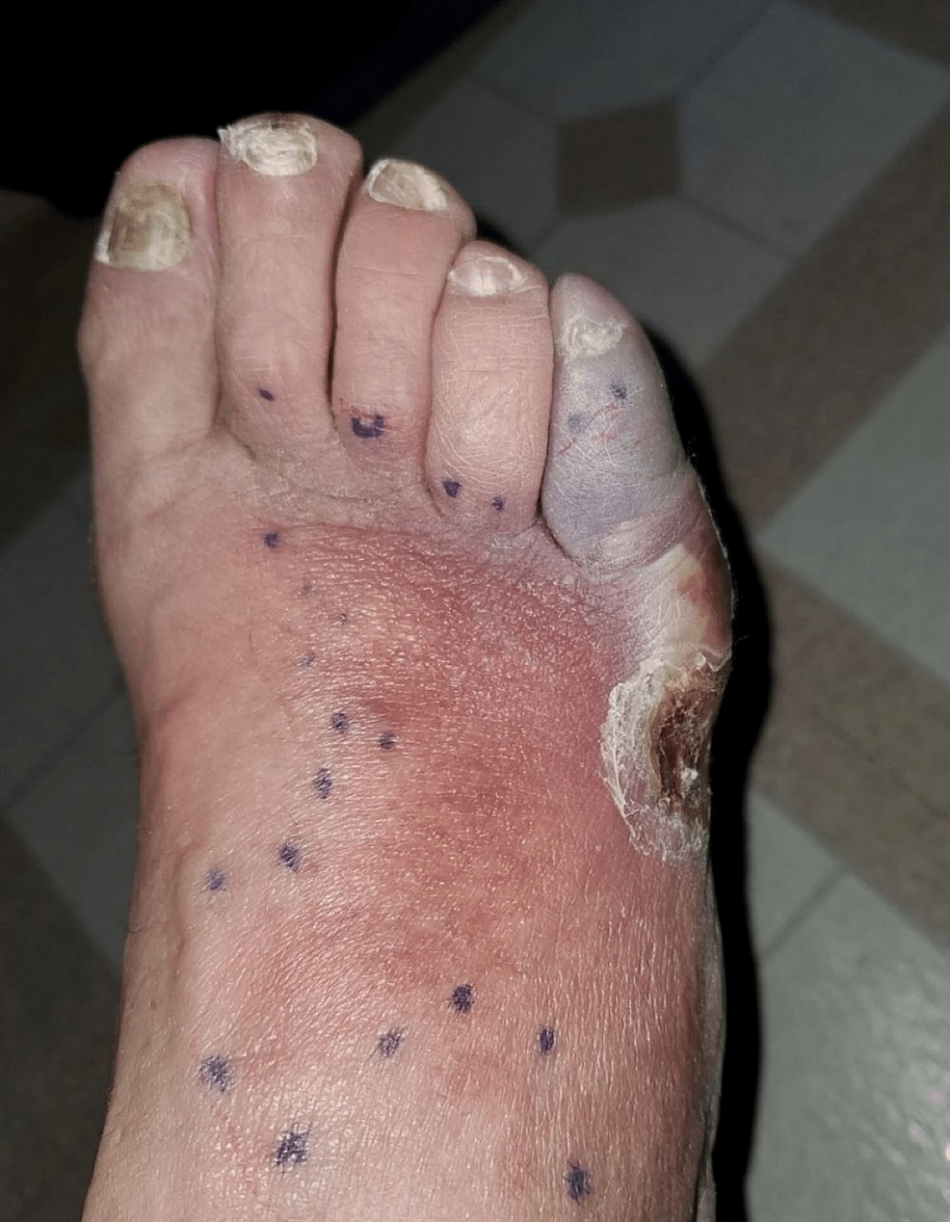
Superior view of the right foot prior to first surgery, fifth toe bluish discoloration, two days following Figure 1.

**Figure 3:**
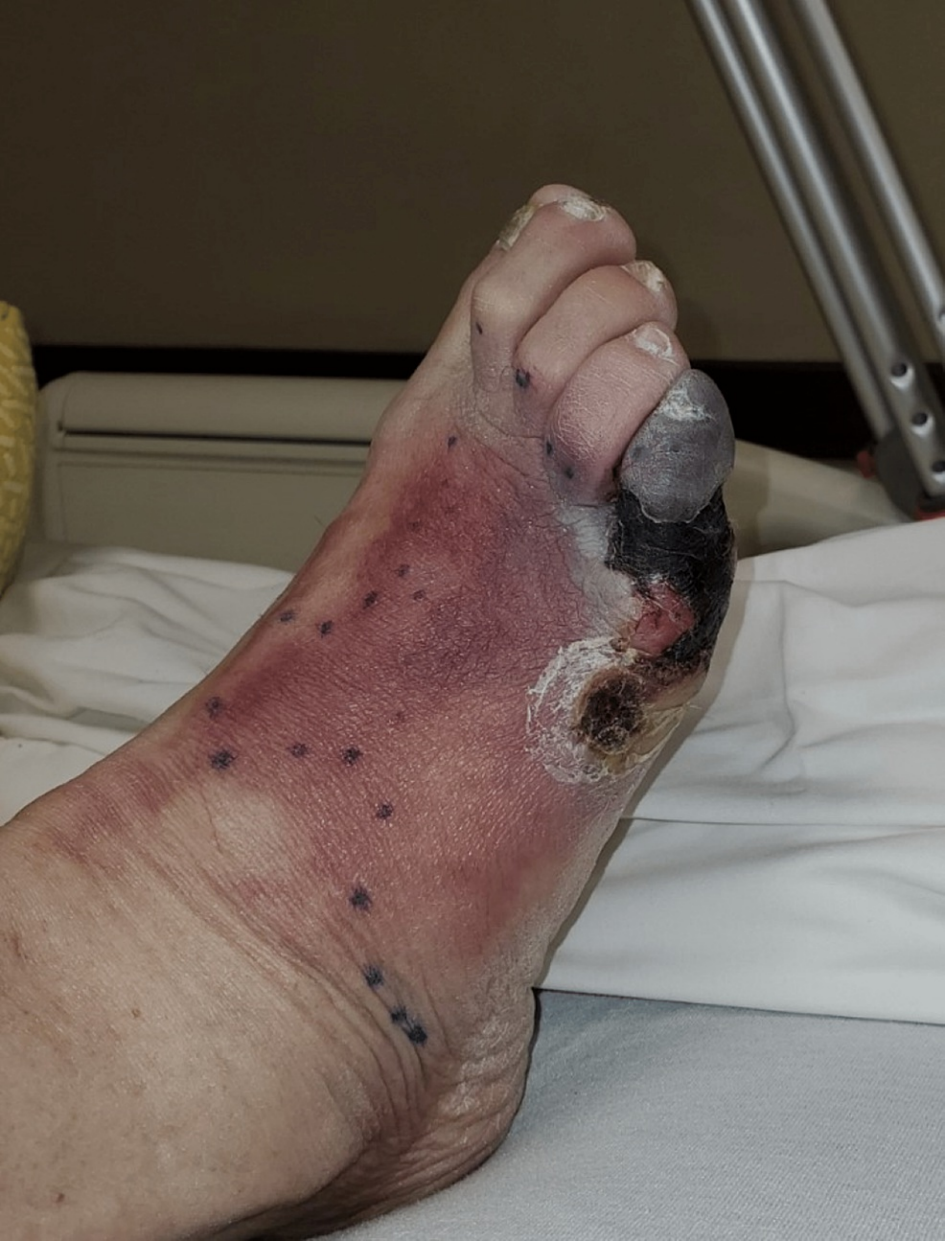
Superior view of the right foot prior to first surgery, fifth toe initial necrosis, two days following Figure 2.

**Figure 4:**
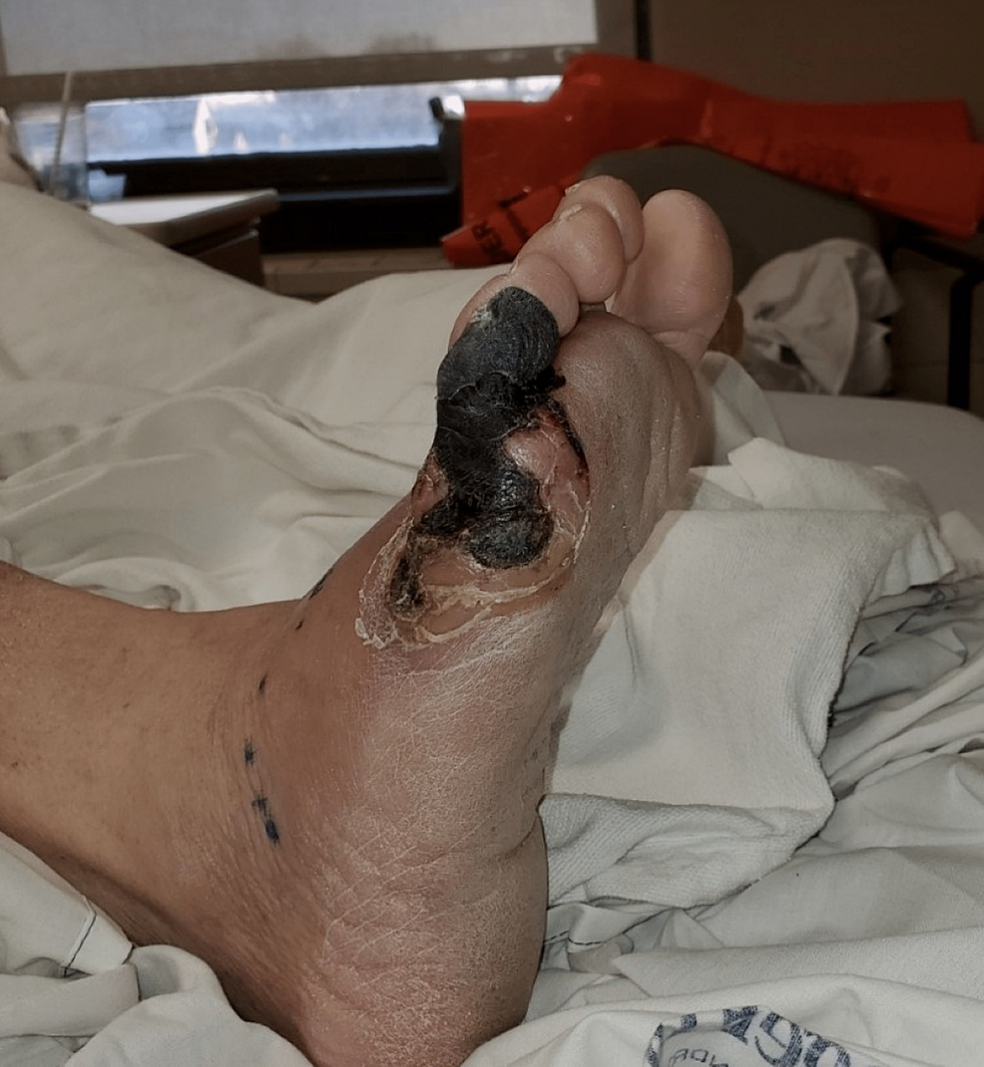
Lateral view of the right foot prior to first surgery, fifth toe complete necrosis, three days following Figure 3.

A 60-year-old male presented to the clinic with a blister over the distal lateral region of the right foot. The patient had a significant history of hypertension, COVID pneumonia, and diabetes mellitus type 2. The patient also had a kidney transplant in 2014. The patient noticed the blister after doing household work and standing on a ladder for a few hours. At that time, there was no visible sign of infection. The patient sought care through his primary care physician and was given metronidazole and dicloxacillin. An X-ray of the right foot was ordered, which showed no signs of osseous involvement. Five days following the initial visit, the patient’s blister was unroofed, and the topical silver gel was prescribed. No sign of an invasive infectious process was noted. Two weeks later, the superficial ulcer became tender to palpation, with noticeable swelling, erythema, and ecchymosis along the lateral border of the fifth metatarsal and fifth metatarsophalangeal joint. The right toe began to show evidence of a worsening infectious process and turned purple. An MRI with and without contrast was ordered to rule out osteomyelitis, and the patient was prescribed trimethoprim-sulfamethoxazole. The MRI results indicated osteomyelitis and gas-forming infection of the fifth toe. The patient was advised to report to the hospital to be admitted for IV antibiotics (daptomycin). A polymerase chain reaction (PCR) test for COVID-19 was negative at that moment. The patient responded poorly to antibiotics which led to the progression of the infection, and ultimately a right fifth toe amputation and fifth metatarsal partial resection was performed with a substitute skin graft. Two days before surgery, a right leg angiogram was performed and showed wide patency with no stenosis of the vessels down into the foot. The evolution from tender to necrotic tissue progressed within seven days (Figures 1-4). All necrotic tissue was debrided before closure and graft placement. The patient was discharged on IV ceftriaxone for six weeks, followed by cefdinir 300 mg bid and linezolid 600 mg bid for two weeks. The patient followed up with wound care three times a week at home.

Two months later, a second surgery was performed due to right foot wound dehiscence, cellulitis, and fourth toe gangrene while undergoing wound care. A right fourth toe amputation with partial metatarsal head resection, a right foot wound secondary closure, and application of a skin graft substitute were performed.

A month later, the patient presented to the hospital with a new-onset redness and swelling of the right foot. Swelling and bluish discoloration of the third right toe and bloody discharge from the wound site were observed. Knowing that most of the time osteomyelitis is polybacterial, wound cultures came back positive for three bacteria including but not limited to methicillin-resistant Staphylococcus aureus (MRSA), Finegolia, and Enterobacter. The positive cultures supported the diagnosis of soft tissue gangrene with concomitant acute osteomyelitis, later confirmed by bone biopsy. Acute osteomyelitis was noted in the second and third toes. The patient had a right trans metatarsal amputation and application of a skin graft substitute to avoid osteomyelitis progression and further surgical interventions. The patient was treated with daptomycin and ertapenem for six weeks. Repeat blood cultures were negative seven days following the surgery.

## Discussion

COVID-19 has presented with various signs and symptoms, including fever, dry cough, tiredness, loss of smell or taste, and, most severely, respiratory failure. It is important to note that the coronavirus can affect multiple organs: the gastrointestinal system, the hematological system, the kidneys, the skin, the eyes, and more [1].

Chilblains, or pernio, is an exaggerated skin response to cold in predisposed individuals. Symptoms typically appear on the hands and feet and are described as pink to violaceous papules. This dermatologic reaction can be idiopathic or associated with many other causes, including autoimmune conditions, genetic mutations, blood-related malignancy, and viral causes, such as Epstein-Barr virus (EBV) [2].

Chilblain-like skin lesions are believed to be associated with COVID-19 due to the increased incidence since the pandemic. It has appeared in warm weather and reported cases have presented with a history of coronavirus infection [3]. Most cases have shown red-purple, red-brown edematous plaques or papules on the toes, soles, and fingers with edema, which is an atypical COVID-19 infection presentation [4-5]. Some cases have shown more unusual skin manifestations such as varicella-like eruptions or livedoid and necrotic lesions [6]. Some patients have had painful, itchy sores or ulcers affecting the extremities. Necrotic lesions are not very common and are mostly seen in elderly patients and those with severe diseases [3].

Since the beginning of the pandemic, the number of diagnoses of Chilblain-like skin lesions, also known as COVID toes, has rapidly increased. It has been reported more frequently in children, teens, and young adults, and yet, the mechanism of this phenomenon remains unclear, although some hypotheses have been formed. It is believed to be linked to microvascular thrombosis due to microangiopathic changes due to an antiviral immune response or systemic inflammation [6-7]. Risk factors that would affect the components of Virchow’s triad are believed to play a role in this phenomenon. Diabetes is known to cause microvascular complications, but the progression is known to be chronic and slow to present [8]. The patient’s COVID toes symptoms showed within two weeks and progressed to gangrene within a month. It would be unlikely that the patient’s diabetes diagnosis would have caused such a rapid progression of the symptoms [9].

There is no established pattern regarding the timing between the active COVID-19 infection and the appearance of the cutaneous symptoms. The symptoms are thought to show late in the infection course [6]. In many cases of COVID toes, evidence of infection has not been consistent or regular when evaluated by polymerase chain reaction for severe acute respiratory syndrome coronavirus 2 (SARS-CoV-2) [6]. The many negative polymerase chain reaction results are believed to be due to the late appearance of the Chilblain-like skin lesions during this phenomenon [3].

There is a similar case of a 47-year-old male who developed gangrene on every toe following COVID-19 pneumonia. The patient had very identical risk factors, such as in this case, diabetes. His COVID toes symptoms progressed to gangrene in a matter of a few days. This patient’s rapid progression of symptoms leads to amputation, requiring bilateral trans metatarsal amputation [9]. Another case series presented two patients with symmetrical peripheral gangrene associated with COVID-19 positive results. The first case was a 37-year-old male without any prior comorbidity, presenting to the emergency room with a week of progressively altered sensorium, fever, and dark discoloration of skin involving the extremities. The second case is a 42-year-old male with a 12-day episode of fever and painful dark discoloration of the toes. This patient had no history of diabetes or hypertension. Sadly, both patients succumbed to their illness [10]. A systematic review was conducted in 2021 by searching PubMed and SCOPUS databases for SARS-CoV-2, COVID-19, and peripheral arterial complications. It found many cases where COVID-19 patients with comorbidities such as hypertension, diabetes, and hypercholesterolemia were more predisposed to develop pain and gangrene. Peripheral gangrene in COVID-19 patients is more likely to happen with prior endothelial dysfunction secondary to hypertension and diabetes. Overall, 30.1% of patients died, and 11.8% required amputations [11].

## Conclusions

The COVID-19 virus has been linked to respiratory complications since the pandemic's beginning. There have been numerous cases associating COVID-19 pneumonia with many vascular complications leading to many amputations. In this case, like many others, there is no evidence that the infection resulting in amputation was caused by the preceding COVID-19 pneumonia, as no PCR test was positive for the virus during the chain of events. The cause is thought to be multifactorial and as mentioned, this patient had many risk factors increasing the probability of this complication. Our case will add to the growing literature on this unexpected association with the eventual goal of early recognition of this possible complication in patients with similar risk factors mentioned previously. Hopefully, enough similar cases could motivate further investigation into the possible need for prophylaxis therapy in selected populations that may be predisposed to this adverse reaction to avoid invasive surgical interventions leaving patients with lifelong residual effects.
